# HIV testing and willingness to get HIV testing at a peer-run drop-in centre for people who inject drugs in Bangkok, Thailand

**DOI:** 10.1186/1471-2458-12-189

**Published:** 2012-03-13

**Authors:** Lianping Ti, Kanna Hayashi, Karyn Kaplan, Paisan Suwannawong, Eric Fu, Evan Wood, Thomas Kerr

**Affiliations:** 1 Faculty of Health Sciences, Simon Fraser University, Burnaby, Canada; 2 British Columbia Centre for Excellence in HIV/AIDS, St. Paul's Hospital, Vancouver, Canada; 3 Interdisciplinary Studies Program, University of British Columbia, Vancouver, Canada; 4 Thai AIDS Treatment Action Group, Bangkok, Thailand; 5 Department of Medicine, University of British Columbia, St. Paul's Hospital, Vancouver, Canada; 6 Urban Health Research Initiative, BC Centre for Excellence in HIV/AIDS, 608-1081 Burrard Street, Vancouver, BC V6Z 1Y6, Canada

**Keywords:** HIV testing, Injection drug use, Thailand, Peer-based interventions

## Abstract

**Background:**

Regular HIV testing among people who inject drugs is an essential component of HIV prevention and treatment efforts. We explored HIV testing behaviour among a community-recruited sample of injection drug users (IDU) in Bangkok, Thailand.

**Methods:**

Data collected through the Mitsampan Community Research Project were used to examine correlates of HIV testing behaviour among IDU and to explore reasons for not being tested. Multivariate logistic regression was used to examine factors associated with willingness to access HIV testing at the drug-user-run Mitsampan Harm Reduction Centre (MSHRC).

**Results:**

Among the 244 IDU who participated in this study, 186 (76.2%) reported receiving HIV testing in the previous six months. Enrolment in voluntary drug treatment (odds ratio [OR] = 2.34; 95% confidence interval [CI]: 1.18 - 4.63) and the tenofovir trial (OR = 44.81; 95%CI: 13.44 - 149.45) were positively associated with having been tested, whereas MSHRC use (OR = 1.78; 95%CI: 0.96 - 3.29) was marginally associated with having been tested. 56.9% of those who had not been tested reported in engaging in HIV risk behaviour in the past six months. 181 (74.2%) participants were willing to be tested at the MSHRC if testing were offered there. In multivariate analyses, willingness to get HIV testing at the MSHRC was positively associated with ever having been to the MSHRC (adjusted odds ratio [AOR] = 2.42; 95%CI: 1.21 - 4.85) and, among females, being enrolled in voluntary drug treatment services (AOR = 9.38; 95%CI: 1.14 - 76.98).

**Conclusions:**

More than three-quarters of IDU received HIV testing in the previous six months. However, HIV risk behaviour was common among those who had not been tested. Additionally, 74.2% of participants were willing to receive HIV testing at the MSHRC. These findings provide evidence for ongoing HIV prevention education, as well potential benefits of incorporating HIV testing for IDU within peer-led harm reduction programs.

## Background

The HIV/AIDS epidemic remains a global challenge, with an estimated 33.3 million people living with HIV globally [1]. In many settings, the fastest growing epidemics of HIV are occurring among people who inject drugs [2-4]. Currently, major public health efforts are underway to scale-up HIV testing [5], as HIV testing leads to early diagnosis, and knowledge of HIV-serostatus may minimize HIV transmission by reducing risky behaviour [6]. Further, testing can help increase access to treatment for injection drug users (IDU) and thereby reduce HIV-related morbidity and mortality, while also bolstering prevention efforts by reducing HIV-I RNA to undetectable levels in infected individuals [7].

Although there is much evidence to support efforts to increase the accessibility of HIV testing among IDU, there are many factors that mitigate the likelihood that IDU will get tested. Barriers such as the fear of an HIV-positive test result, HIV-related stigma, and the resulting impacts on relationships with family and public authorities are among the reasons why IDU do not get tested for HIV [8,9]. In Thailand, where the prevalence of HIV among IDU is as high as 50% [10], an excessive focus on drug law enforcement has likely contributed to the suboptimal coverage of HIV prevention, treatment and care among this population [11,12]. For example, some public hospitals reportedly collect and share information concerning patients' drug use behaviour with police in Thailand [11]. This compromising of patient confidentiality prevents many IDU from wanting to use government testing services, especially given that, in order to qualify for a free HIV test at public testing clinics, IDU must declare their risk behaviour (P. Sririrund, personal communication, April 30, 2011). In addition, if IDU were to get HIV testing at private clinics, there would be the burden of the cost of an HIV test imposed upon them. Collectively, these barriers could potentially undermine access to HIV testing among this population. Recent studies have found that uptake of HIV testing among IDU in parts of Asia is as low as 20% annually [13].

Several studies have shown that peer-based interventions for drug users can often extend the reach and effectiveness of conventional public health programs [14,15], especially in settings where drug use is heavily penalized, as in Thailand [16]. There has been increasing interest in novel, low-threshold methods of HIV testing, including peer-based testing [17,18]. However, there are currently no peer-based models of HIV testing in Thailand, and concerns have been expressed regarding the inadequate voluntary counselling and HIV testing services offered to IDU in this setting [11]. Given that knowledge of HIV-positive serostatus can reduce the health burden associated with HIV/AIDS among IDU, and HIV testing and counselling continue to be supported by international health organizations globally [19,20], the lack of these services in Thailand has prompted calls for increased voluntary counselling, testing and information services across the country [11].

According to the Reference Group to the United Nations on HIV and Injecting Drug Use, there are an estimated 160,000 IDU living in Thailand [2], of whom the majority are male and between the ages of 30-40 years [16,21]. While there exists some evidence indicating why individuals engaging in illicit drug use have low uptake of HIV testing services [21], little is known about the factors that influence HIV testing among IDU in Thailand. Therefore, we sought to investigate the prevalence and correlates of HIV testing behaviour among a community-recruited sample of IDU in Bangkok, Thailand. As well, given the growing interest in low-threshold and peer-based HIV testing programs, we also sought to assess willingness to get tested for HIV at a drug-user-run drop-in centre in Bangkok.

## Methods

The Mitsampan Community Research Project (MSCRP) is a collaborative research project involving the Mitsampan Harm Reduction Centre (Bangkok, Thailand), the Thai AIDS Treatment Action Group (Bangkok, Thailand), Chulalongkorn University (Bangkok, Thailand) and the British Columbia Centre for Excellence in HIV/AIDS (Vancouver, Canada). During June and July of 2009, the research partners undertook a cross-sectional study involving 317 community-recruited IDU. Potential participants were recruited through peer-based outreach efforts and word of mouth. Study participants were then invited to attend the MSHRC to participate in the study. All participants provided informed consent and completed an interviewer-administered questionnaire eliciting information about demographic characteristics, drug use, HIV risk behaviour, criminal justice system exposure, and experiences with health care. All participants were given 350 baht (approximately US$11) upon completion of the questionnaire. The study has been approved by the research ethics boards of the University of British Columbia and Chulalongkorn University.

For the present analyses, we restricted the study sample to individuals who were HIV-negative or of unknown HIV serostatus. As a first step, we compared IDU who had and had not been tested for HIV in the past six months using the Pearson χ^2 ^test and Fisher's exact test (when one or more cells contained values less than or equal to five). Among the participants who had not been tested for HIV in the past six months, we examined the prevalence of ongoing HIV risk behaviour and asked them to indicate reasons why they had not been tested. Second, we compared IDU who were and were not willing to get free HIV testing at MSHRC, again using the Pearson χ^2 ^test and Fisher's exact test (when one or more cells contained values less than or equal to five). The "willingness to get free HIV testing at MSHRC" variable referred to whether participants would get tested at least once at the peer-run drop-in centre. Specifically, we asked: "If the MSHRC offered free HIV testing with pre- and post-counselling (including referrals to clinics/hospitals), would you take the test?" We then built a multivariate logistic regression model to identify independent predictors of willingness to get free HIV testing at MSHRC by including all variables that were associated with the outcome at the *p *≤ 0.10 level in bivariate analyses.

Variables considered in both initial bivariate analyses included: gender, median age, relationship status (married/common law, regular partner vs. separated, dating, single), education level (≥ secondary school vs. < secondary school), heroin injection in the past six months (> once per week vs. ≤ once per week), midazolam injection in the past six months (> once per week vs. ≤ once per week), methamphetamine injection in the past six months (> once per week vs. ≤ once per week), lent or borrowed syringes to/from others in the past six months (yes vs. no), enrolled in voluntary drug treatment in the past six months (yes vs. no), had unprotected sex in the past six months (yes vs. no), incarceration in the past six months (yes vs. no), reporting barriers to accessing health services (any barriers vs. no barriers), and having ever been to MSHRC (yes vs. no). Our barriers to testing variable included the following: limited hours of operation, long wait times, didn't know where to go, jail, detention, prison, no identification, identification registered somewhere else, no money, was treated poorly by healthcare professionals, fear of sharing information of drug using status with the police and/or narcotics control board, difficulty keeping appointments, transportation, and others. Additionally, because Bangkok was a site of the tenofovir trial (a pre-exposure prophylaxis trial) that included IDU, being enrolled in the tenofovir trial (yes vs. no) was also considered in the first bivariate analyses focused on HIV testing behaviour in the past six months. All *p*-values were two-sided.

## Results

In total, 244 individuals who were HIV-negative or of unknown HIV serostatus were included in the study, including 77 (31.6%) females. The median age of the participants at the time of interview was 37 years (range: 20 - 72 years). In total, 186 (76.2%) participants reported that they had been tested for HIV in the past six months. As indicated in Table 1, results show that being enrolled in voluntary drug treatment in the past six months (odds ratio [OR] = 2.34; 95% confidence interval [CI]: 1.18 - 4.63) and being in the tenofovir trial (OR = 44.81; 95% CI: 13.44 - 149.45) were positively associated with having been tested for HIV. As well, having been to MSHRC (OR = 1.78; 95%CI: 0.96 - 3.29) was marginally associated with having been tested for HIV. Although the odds ratio for the incarceration variable could not be calculated because one cell contained a zero, there was a trend towards a positive association between having been incarcerated in the past six months and having been tested for HIV. Of the 58 participants who were not tested for HIV, 41 responded to the question: "Why haven't you taken an HIV test?" Of these, 27 (65.9%) stated that they believed they were HIV-negative. However, 33 (56.9%) participants who did not get an HIV test engaged in at least one of the following risky behaviours in the past six months: syringe borrowing, syringe lending, or unprotected vaginal or anal intercourse.

**Table 1:** Bivariate analyses of factors associated with being tested for HIV in the past six months among IDU in Bangkok, Thailand (n = 244)

	Tested for HIV in past six months n (%)			
**Characteristic**	**Yes** **186 (76.2%)**	**No** **58 (23.8%)**	**Odds Ratio(95% CI)**	***p - *value**

**Gender**				

Female	54 (29.0)	23 (39.7)	0.62 (0.34 - 1.15)	0.13

Male	132 (71.0)	35 (60.3)		

**Median age**				

≥ 37 years old	96 (51.6)	24 (41.4)	1.51 (0.83 - 2.74)	0.17

< 37 years old	90 (48.4)	34 (58.6)		

**Relationship status**				

Married/common law, regular partner	100 (53.8)	33 (56.9)	0.88 (0.49 - 1.60)	0.68

Separated, dating, single	86 (46.2)	25 (43.1)		

**Education level**				

≥ secondary education	66 (35.5)	23 (39.7)	0.84 (0.46 - 1.53)	0.56

< secondary education	120 (64.5)	35 (60.3)		

Heroin injection*				

> once per week	54 (29.0)	17 (29.3)	0.99 (0.52 - 1.89)	0.97

≤ once per week	132 (71.0)	41 (70.7)		

Midazolam injection*				

> once per week	114 (61.3)	32 (55.2)	1.29 (0.71 - 2.33)	0.41

≤ once per week	72 (38.7)	26 (44.8)		

Methamphetamine injection*				

> once per week	71 (38.2)	18 (31.0)	1.37 (0.73 - 2.58)	0.32

≤ once per week	115 (61.8)	40 (69.0)		

Lent or borrowed syringes to/from others*				

Yes	33 (17.7)	9 (15.5)	1.17 (0.53 - 2.62)	0.70

No	153 (82.3)	49 (84.5)		

Enrolled in voluntary drug treatment*				

Yes	75 (40.3)	13 (22.4)	2.34 (1.18 - 4.63)	0.01

No	111 (59.7)	45 (77.6)		

Had unprotected sex*				

Yes	87 (46.8)	29 (50.0)	0.88 (0.49 - 1.59)	0.67

No	99 (53.2)	29 (50.0)		

Incarceration*				

Yes	11 (5.9)	0 (0.0)	--	0.07

No	175 (94.1)	58 (100.0)		

**Reporting barriers to accessing health services**				

Any barriers	68 (36.6)	21 (36.2)	1.02 (0.55 - 1.87)	0.96

No barriers	118 (63.4)	37 (63.8)		

**Ever been to MSHRC**				

Yes	90 (48.4)	20 (34.5)	1.78 (0.96 - 3.29)	0.06

No	96 (51.6)	38 (65.5)		

**In tenofovir trial**				

Yes	132 (71.0)	3 (5.2)	44.81 (13.44 - 149.45)	< 0.01

No	54 (29.0)	55 (94.8)		

* Activities in the previous six months

*IDU *Injecting drug users

*MSHRC *Mitsampan Harm Reduction Centre

*CI *Confidence Interval

In total, 181 (74.2%) participants responded that they were willing to get tested at the MSHRC. As shown in Table 2, factors positively associated with a willingness to be tested for HIV at the MSHRC included being enrolled in voluntary drug treatment in the past six months (OR = 1.94; 95%CI: 1.02 - 3.68), reporting a barrier to accessing health services (OR = 1.99; 95%CI: 1.05 - 3.77), and having been to the MSHRC (OR = 3.17; 95%CI: 1.68 - 6.01). There was also a marginally significant association between willingness to get tested at the MSHRC and having lent or borrowed syringes to/from others in the past six months (OR = 2.36; 95%CI: 0.94 - 5.90). Additionally, female sex was negatively associated with willingness to be tested for HIV at the MSHRC (OR = 0.46; 95%CI: 0.26 - 0.84).

**Table 2:** Bivariate analyses of factors associated with willingness to get free HIV testing at the MSHRC among IDU in Bangkok, Thailand (n = 244)

	HIV testing at MSHRC n (%)			
**Characteristic**	**Yes** **181 (74.2%)**	**No** **63 (25.8%)**	**Odds Ratio(95% CI)**	***p - *value**

**Gender**				

Female	49 (27.1)	28 (44.4)	0.46 (0.26 - 0.84)	0.01

Male	132 (72.9)	35 (55.6)		

**Median age**				

≥ 37 years old	92 (50.8)	28 (44.4)	1.29 (0.73 - 2.30)	0.38

< 37 years old	89 (49.2)	35 (55.6)		

**Relationship status**				

Married/common law, regular partner	103 (56.9)	30 (47.6)	1.45 (0.82 - 2.58)	0.20

Separated, dating, single	78 (43.1)	33 (52.4)		

**Education level**				

≥ secondary education	68 (37.6)	21 (33.3)	1.20 (0.66 - 2.20)	0.55

< secondary education	113 (62.4)	42 (66.7)		

Heroin injection*				

> once per week	56 (30.9)	15 (23.8)	1.43 (0.74 - 2.77)	0.28

≤ once per week	125 (69.1)	48 (76.2)		

Midazolam injection*				

> once per week	113 (62.4)	33 (52.4)	1.51 (0.85 - 2.69)	0.16

≤ once per week	68 (37.6)	30 (47.6)		

Methamphetamine injection*				

> once per week	70 (38.7)	19 (30.2)	1.46 (0.79 - 2.70)	0.23

≤ once per week	111 (61.3)	44 (69.8)		

Lent or borrowed syringes to/from others*				

Yes	36 (19.9)	6 (9.5)	2.36 (0.94 - 5.90)	0.06

No	145 (80.1)	57 (90.5)		

Enrolled in voluntary drug treatment*				

Yes	72 (39.8)	16 (25.4)	1.94 (1.02 - 3.68)	0.04

No	109 (60.22)	47 (74.6)		

Had unprotected sex*				

Yes	86 (47.5)	30 (47.6)	1.00 (0.56 - 1.77)	0.99

No	95 (52.5)	33 (52.4)		

Incarceration*				

Yes	10 (5.5)	1 (1.6)	3.63 (0.45 - 28.91)	0.30

No	171 (94.5)	62 (98.4)		

**Reporting barriers to accessing health services**				

Any barriers	73 (40.3)	16 (25.4)	1.99 (1.05 - 3.77)	0.03

No barriers	108 (59.7)	47 (74.6)		

**Ever been to MSHRC**				

Yes	94 (51.9)	16 (25.8)	3.17 (1.68 - 6.01)	< 0.01

No	87 (48.1)	47 (74.6)		

* Activities in the previous six months

*IDU *Injecting drug users

*MSHRC *Mitsampan Harm Reduction Centre

*CI *Confidence Interval

Table 3 presents the results of multivariate analyses of factors associated with willingness to get HIV testing at the MSHRC. As indicated here, having been to the MSHRC (adjusted odds ratio [AOR] = 2.42; 95%CI: 1.21 - 4.85) remained positively associated with willingness to get HIV testing at the peer-run drop-in centre. An analysis of interactions was then conducted between gender and enrolment in voluntary drug treatment in the past six months. A positive association between willingness to be tested at the MSHRC and being enrolled in voluntary drug treatment was found for females (AOR = 9.38; 95%CI: 1.14 - 76.98), while a marginal positive association between willingness to get tested for HIV at the MSHRC and non-enrolment in voluntary drug treatment was found for males (AOR = 2.07; 95%CI: 0.98 - 4.39).

**Table 3:** Multivariate logistic regression analyses of factors associated with willingness to get free HIV testing at MSHRC among IDU in Bangkok, Thailand (n = 244)

Variable	Adjusted Odds Ratio (AOR)	95% Confidence Interval (CI)	*p - *value
Lent or borrowed syringes to/from others*			

(yes vs. no)	1.49	(0.56 - 4.00)	0.43

**Reporting barriers to accessing health services**			

(yes vs. no)	1.22	(0.59 - 2.51)	0.58

**Ever been to MSHRC**			

(yes vs. no)	2.42	(1.21 - 4.85)	0.01

Gender (not enrolled in voluntary treatment*)			

(male vs. female)	2.07	(0.98 - 4.39)	0.06

Gender (enrolled in voluntary treatment*)			

(male vs. female)	0.22	(0.03 - 1.85)	0.16

Enrolled in voluntary drug treatment* (male)			

(yes vs. no)	1.01	(0.46 - 2.18)	0.99

Enrolled in voluntary drug treatment* (female)			

(yes vs. no)	9.38	(1.14 - 76.98)	0.04

*Activities in the previous six months

*IDU *Injection Drug Users

## Discussion

In the present study, we found that HIV testing was common among Thai IDU, with 76.2% reporting that they had been tested in the past six months. Having been enrolled in voluntary drug treatment and being enrolled in the tenofovir trial was positively associated with having been tested for HIV, while having been to the MSHRC was marginally associated. Among those who had not been tested for HIV in the past six months, approximately 56% had recently engaged in some form of HIV risk behaviour. We also found that almost three-quarters of the sample (74.2%) expressed willingness to get tested for HIV at the drug-user-run MSHRC. In a multivariate analysis, having been to MSHRC was independently and positively associated with willingness to get tested at MSHRC. Enrolment in voluntary drug treatment was also associated with willingness to get tested at the MSHRC, although this relationship interacted with gender, with women in treatment being the more willing to get tested at the MSHRC.

We found that just over three-quarters (76.2%) of our sample of IDU had previously been tested for HIV in the past six months. Our findings are consistent with previous studies demonstrating fairly high levels of HIV testing in other middle-income settings contending with high rates of HIV infection among IDU, including Andhra Pradesh, India (89%) [22] and St. Petersburg, Russia (76%) [23]. However, our findings are inconsistent with previous studies that show low uptake of HIV testing among IDU in Asia [13]. That said, it is unclear whether the high rate of testing observed here is partially a reflection of the existence of the tenofovir trial, as 55% of participants had enrolled in this study. Although HIV testing appears to be quite common among IDU in Bangkok, there were still a number of active drug-using participants who had not been tested in the past six months. Among the participants who were not tested for HIV, the majority perceived themselves to be HIV-negative even though they had engaged in at least one HIV risk behaviour in the past six months. This raises concern that some IDU in Thailand may be unaware of their HIV risk, indicating a need for intensified and targeted outreach, education and testing efforts to reach these individuals [9,24]. Interestingly, our findings reveal that the type of illicit drugs injected (heroin, midazolam, and methamphetamine) was not associated with either of our outcome variables, suggesting that our findings are uniform across individuals who use different types of drugs.

UNODC recommends community-based, voluntary drug treatment programs across South East Asia as a substitute for incarceration and compulsory drug detention centres for IDU [25]. Voluntary drug treatment programs, in particular opiate substitution therapy (OST), have been previously associated with a reduction in risky behaviour and HIV infection among IDU in various settings [26-28]. Adding to the benefits of voluntary treatment, we found that participants who were enrolled in voluntary treatment were significantly more likely to get tested for HIV compared to those who were not enrolled in voluntary treatment. Our findings support the recommendation of the United States Centres for Disease Control and Prevention (US CDC) to integrate HIV testing services as part of voluntary drug treatment [29]. However, concerns have been raised about the nature of current HIV testing in voluntary drug treatment centres in Thailand, as it has been reported that in some of these centres, HIV testing is mandatory and a condition of receiving services [11]. Given that our study did not differentiate between mandatory and voluntary HIV testing in voluntary treatment programs, future research should seek to untangle this complex relationship. Nevertheless, in these settings, replacing the system of mandatory testing with voluntary HIV testing in these treatment programs may prove to be effective in increasing the proportion of IDU in Thailand who get tested, thereby allowing for early diagnosis, enhanced access to antiretroviral therapy (ART) treatment, and targeted education and interventions to control the spread of HIV [6].

In addition to being enrolled in voluntary drug treatment, being enrolled in the tenofovir trial was also positively associated with HIV testing among IDU. The tenofovir trial in Thailand, sponsored by the US CDC, was launched in 2005 in an attempt to examine the safety and efficacy of this antiretroviral drug. Currently, tenofovir (alone) is being provided to approximately 2,400 HIV-negative IDU and 17 drug treatment clinics across Bangkok [30]. Results of the US CDC-sponsored pre-exposure prophylaxis trial in Thailand are to be revealed in early 2012 [31]. The increased odds of HIV testing among IDU in the tenofovir trial could be attributed to the fact that all participants in the trial receive free rapid HIV testing on an ongoing basis. Although our findings show a positive association between being enrolled in the tenofovir trial and HIV testing, concerns have been expressed over the fact that the trial sites in Thailand failed to provide sterile syringes and needles to IDU participants [32,33]. Efforts to engage and consult with IDU community groups at an earlier stage of the trial design process may have helped alleviate these concerns and should be considered for future HIV prevention trials as a means of ensuring appropriate access to HIV prevention technologies among trial participants.

Internationally, peer outreach and peer-run initiatives have been shown to be successful in extending the reach and effectiveness of conventional public health programs, including those focused on preventing and treating HIV/AIDS among IDU [14,34,35]. Since the launch of Thailand's 2003 "War on Drugs" campaign, the government has continued to rely on repressive approaches to drugs, including arbitrary arrests, blacklisting, drug planting by police, extrajudicial executions and other human rights violations of people involved with drugs [11,36,37]. In this context, severe stigma and discrimination persist against Thai IDU, prompting many to avoid public health programs [37]. Additionally, problems related to stigmatizing attitudes of health care providers focused on IDU have been reported among Thai health professionals, including nursing students, which in turn likely adversely affects the willingness of IDU to access health care [38]. Given these problems, the noted effectiveness of various peer-based methods for IDU, and the high willingness to access HIV testing at the MSHRC, peer-based HIV testing interventions for IDU may have high potential for success in this setting. In the present study, having been to the MSHRC previously was significantly and positively associated with willingness to get HIV testing at the centre. In an earlier study conducted on the MSHRC, results showed that the main reason IDU did not access the centre was the lack of knowledge of its existence [16]. Therefore, future efforts should focus on increasing awareness of and improving access to the MSHRC and other drop-in centres like it. Since the focus of this paper was on willingness to get HIV testing at the MSHRC without specifying whether the testing was peer-delivered, future research efforts should seek to determine whether IDU would be willing to be tested by a peer either in the context of a drug-user-run harm reduction program or in other conventional health care settings.

Enrolment in voluntary drug treatment was also associated with willingness to get HIV testing at the MSHRC. As mentioned above, there was an interaction effect involving enrolment in voluntary treatment and gender, and willingness to get tested at the MSHRC. Among participants who were not enrolled in voluntary treatment, males were significantly more willing than females to get tested at the MSHRC, although this association did not reach conventional significance. Among female IDU, those engaged in treatment were significantly more willing to get tested at the MSHRC compared to those out of treatment. In light of these findings, future research should seek to unpack the gender dynamics surrounding addiction treatment enrolment and HIV testing behaviour within this setting.

This study has several limitations. First, because of the cross-sectional nature of the study, there is an inability to determine a temporal relationship between exposure and outcome, and therefore causation cannot be inferred. Second, the data collected were self-reported and may be subject to reporting biases. Socially desirable reporting as well as recall bias may affect reports of both risk behaviour and testing behaviour. Third, since the study sample was small and not randomly selected, the study may not be representative of Thai IDU. As well, our findings may not be generalizable to other populations of IDU. Lastly, because of the small sample size, there were wide intervals around some of the estimates reported.

## Conclusions

In the present study, we found high rates of HIV testing among Thai IDU. However, we found that among participants who were not tested for HIV, a high proportion had recently engaged in some form of HIV risk behaviour. We also observed a high rate of willingness to get free HIV testing at the MSHRC. Willingness to be tested was independently associated with having been to MSHRC, and among women, having been enrolled in voluntary drug treatment was also associated with willingness to be tested at the MSHRC. These findings highlight the need for ongoing educational efforts related to HIV transmission, as well opportunities to expand harm reduction strategies to include peer-led HIV testing for IDU in Thailand.

## Competing interests

The authors declare that they have no competing interests.

## Authors' contributions

LT and TK designed the study. EF conducted the statistical analyses. LT drafted the manuscript and incorporated all suggestions from all the co-authors. All authors made significant contributions to the conception of the analyses, interpretation of the data, and drafting of the manuscript. All authors read and approved the final manuscript.

## Pre-publication history

The pre-publication history for this paper can be accessed here:

http://www.biomedcentral.com/1471-2458/12/189/prepub
